# An Examination of Differences in Psychological Resilience between Social Anxiety Disorder and Posttraumatic Stress Disorder in the Context of Early Childhood Trauma

**DOI:** 10.3389/fpsyg.2017.02058

**Published:** 2017-12-11

**Authors:** Melanie Marx, Susanne Y. Young, Justin Harvey, David Rosenstein, Soraya Seedat

**Affiliations:** ^1^Psychiatry, Faculty of Medicine and Health Sciences, Stellenbosch University, Cape Town, South Africa; ^2^Department of Statistics and Actuarial Science, Faculty of Economics and Management Sciences, Stellenbosch University, Stellenbosch, South Africa

**Keywords:** social anxiety disorder, posttraumatic stress disorder, physical neglect, physical abuse, emotional abuse, sexual abuse, childhood trauma, resilience

## Abstract

**Background:** Much of the research on anxiety disorders has focused on associated risk factors with less attention paid to factors such as resilience that may mitigate risk or offer protection in the face of psychopathology.

**Objective:** This study sought to compare resilience in individuals with posttraumatic stress disorder (PTSD) and social anxiety disorder (SAD) relative to age-, gender- and education- matched individuals with no psychiatric disorder. We further assessed the correlation of resilience scores with childhood trauma severity and type.

**Method:** The sample comprised of 93 participants, 40 with SAD with childhood trauma), 22 with PTSD with childhood trauma, and 31 with no psychiatric disorder (i.e., healthy matched controls). Participants were administered the Mini-International Neuropsychiatric Interview (MINI), Liebowitz Social Anxiety Scale (LSAS), Clinician-Administered PTSD Scale (CAPS), Childhood Trauma Questionnaire—Short Form (CTQ-SF), and the Connor-Davidson Resilience Scale (CD-RISC). The mean age of participants was 34 years (*SD* = 11). 52 Participants were female (55.9%) and 54 Caucasian (58.1%). Analysis of variance was used to assess for significant group differences in resilience scores. Non-parametric correlation analyses were conducted for resilience and different types of childhood trauma.

**Results:** There were significant differences in resilience between the SAD and PTSD groups with childhood trauma, and controls. Both disorder groups had significantly lower levels of resilience than healthy controls. No significant correlation was found between total resilience scores and childhood trauma scores in the childhood trauma (SAD and PTSD) groups. However, in the combined dataset (SAD, PTSD, healthy controls), significant negative correlations were found between resilience scores and emotional abuse, emotional neglect, and total childhood trauma scores.

**Conclusions:** Patients who have PTSD and SAD with childhood trauma appear to be significantly less resilient than those with no disorder. Assessing and addressing resilience in these disorders, particularly when childhood trauma is present, may facilitate long-term recovery and warrants further investigation.

## Introduction

Childhood trauma is a significant etiological precursor for the development of psychopathology (Kendler et al., 1995; Agid et al., 2000; Heim and Nemeroff, 2001; Spila et al., 2008). Childhood trauma is defined as any traumatic experience occurring before the age of 18 and is categorized into five subtypes, namely emotional abuse, emotional neglect, physical abuse, physical neglect, and sexual abuse (Bernstein et al., 1997). Several retrospective studies indicate that early exposure to trauma is associated in adulthood with an increased risk of developing anxiety and stress-related disorders (Kendler et al., 1995; Agid et al., 2000; Heim and Nemeroff, 2001; Spila et al., 2008), including posttraumatic stress disorder (PTSD) (Ballenger et al., 2004; Bandelow et al., 2004; Etkin and Wager, 2007; Yehuda et al., 2010) and social anxiety disorder (SAD), (Heim and Nemeroff, 2001; Fossion et al., 2014), and an elevated stress response to mild stressors (Nemeroff, 2004). Norman et al. (2012) documented a significant association between childhood physical abuse, and emotional abuse and neglect, and anxiety disorders; see Carr et al. (2013) for a review on the associations between childhood abuse (physical abuse and neglect, emotional abuse and neglect, sexual abuse) and anxiety disorders. Specifically, a significant relationship has been observed between physical abuse (Norman et al., 2012) and sexual abuse in childhood and PTSD (Kendler et al., 1995). In addition, multiple trauma exposures in early life have been linked to increased PTSD symptoms (Collin-Vézina et al., 2011). Suliman et al. (2009) found that adolescents who experienced numerous childhood adversities were more likely to experience increased severity of their PTSD symptoms. In adults, Simon et al. (2009) documented greater disorder severity in individuals with the generalized subtype of SAD who also had childhood maltreatment.

However, not all individuals who experience childhood adversities develop PTSD, SAD or other psychopathology as adults (Collishaw et al., 2007) and may be regarded as possessing resilience characteristics that protect them from developing psychopathology. This resilience may present as capacities that engender adaptation after trauma exposure. Resilience lies on a continuum ranging from well-adapted (and highly resilient) to maladapted (and prone to psychiatric disorders) (Ehlert, 2013). Resilience is the ability to cope after a trauma/stressor (Masten et al., 1999; Masten, 2001; Connor and Davidson, 2003; Tusaie and Dyer, 2004). Resilience is further defined as a set of individual characteristics that may offer coping/protection when faced with trauma (Hoge et al., 2007). These characteristics include a certain way of perceiving, or a pattern of thinking and/or decision making, in reaction to different traumas (Agaibi and Wilson, 2005). Several factors have been associated with resilience, namely an internal locus of control, a sense of meaning, a strong self-esteem, and good problem-solving skills (Rutter, 1985; Taylor et al., 2000; Masten et al., 2009).

In this paper we refer to resilience as a measure of the ability to cope after a trauma/stressor, as described by Connor and Davidson (2003). The Connor-Davidson Resilience Scale (CD-RISC) assesses personal competence, high standards, and instincts; capability of enduring negative emotional experiences; positive acceptance of change and secure relationships; control and spiritual influences (Connor and Davidson, 2003). A trauma or stressor may disrupt the biopsychospiritual homeostasis of an individual. Connor and Davidson (2003) explain that there can be four reactions to a trauma/stressor. First, the trauma/stressor may present the person with an opportunity to grow, increase their resilience, and thereby facilitate return to a higher level of homeostasis. Second, the person may move forward and slightly beyond the trauma/stressor, resulting in a return to baseline homeostasis (as it was before the trauma/stressor). Third, the person may overcome the stressor but return to a lower level of homeostasis. Lastly, the person may maladjust and use destructive means to cope with the trauma/stressor (Connor and Davidson, 2003). The recent shift in focus away from pathology and problem orientation to health promotion has resulted in more awareness of the importance of resilience to wellness (Bonanno, 2004).

Simon et al. (2009) hypothesized that childhood trauma (as measured with the total CTQ score) may be associated with less resilience. Specifically, emotional neglect was associated with less resilience in adults with SAD (Simon et al., 2009). Simeon et al. (2007) found that childhood trauma was significantly negatively correlated with resilience in healthy adults. However, contrary to these findings, DuMont et al. (2007) assessed, among other variables, childhood abuse, childhood neglect and resilience in 676 adults and found that individuals who had experienced adversities and stressful life events were more resilient. This is supported by other studies that have shown that in the face of childhood trauma, including childhood sexual abuse (CSA), some individuals are able to positively adapt and grow (Luther and Cicchetti, 2000; Bonanno, 2004; Wright et al., 2005). Valentine and Feinauer (1993) assessed 57 women who had experienced CSA but were functioning well as adults. Factors that contributed to their well-being as adults included the ability to see that they were the victims, not feeling responsible for the abuse, and developing a sense of purpose. These findings underscore the importance of examining resilience in relation to childhood trauma in the etiology of SAD. In a study by Himelein and McElrath (1996), no differences were documented between a traumatized group who experienced CSA and non-victimized controls, in terms of the overall level of adjustment. In addition, Rutter (2007) suggested that childhood trauma may increase resilience in adulthood, even in the presence of a mental disorder. This phenomenon can be explained by “stress innoculation theory” which posits that a toughening in physiology occurs (Rutter, 1987), with the experience of childhood trauma in some individuals contributing to their being less affected by stressors in adulthood (Campbell-Sills et al., 2006). It has also been found that increased traits of resilience may ameliorate PTSD symptoms, in individuals who have suffered childhood abuse and neglect (Fincham et al., 2009). In addition, a study by Campbell-Sills et al. (2006) found those individuals who had higher levels of childhood emotional neglect also showed greater symptom severity, but only if they were less resilient. In other words, of all participants, those who reported high levels of childhood emotional neglect and high levels of resilience, scored lowest on psychiatric symptoms (Campbell-Sills et al., 2006).

Among South Africans, anxiety disorders are the most prevalent of lifetime mental disorders at 15.8%, with SAD and PTSD at 2.8 and 2.3%, respectively (Herman et al., 2009). Resilience may be a valuable tool in the treatment of adult individuals with anxiety (Connor and Davidson, 2003) and especially in those who have faced past trauma. In addition, Collishaw et al. (2007) have suggested that our understanding of how abuse affects psychosocial development may be enhanced by our understanding of resilience. Furthermore, a systematic review on child abuse and neglect concluded that more studies should research elements that may contribute to resilience (Kaplan et al., 1999). There has been little investigation of resilience in adults with an anxiety disorder and early childhood trauma exposure, moreover in SAD and PTSD. It is plausible that childhood trauma may interact with the degree of resilience in adult individuals with PTSD and SAD. Resilience, as measured by the CD-RISC, has been shown to mitigate depressive symptom severity in adults, as contributed to by childhood abuse and other traumas (Wingo et al., 2010). However, to our knowledge there are no studies that have assessed resilience in PTSD and SAD in the context of childhood trauma. This is important given that resilience, against a background of childhood trauma, may be a modifiable target for treatment recovery in patients with anxiety disorders.

We hypothesized that there would be significant differences in resilience among adults with PTSD secondary to childhood trauma, adults with SAD and childhood trauma, and a control group without childhood trauma, PTSD or SAD. Second, we hypothesized that resilience scores would be negatively correlated with childhood trauma scores (physical and emotional neglect and abuse, and sexual abuse) in adults with PTSD and SAD. Thirdly, when combining all groups (PTSD, SAD, and controls), we hypothesize that due to the inclusion of the control group in this analyses, a negative relationship would be found between different childhood traumas and resilience.

## Method

### Design

We undertook a cross-sectional analysis of data from a larger imaging-genetics study of SAD and PTSD.

### Participants

Participants were recruited from community clinics, hospitals, non-governmental organizations (NGOs) and other psychiatric institutions in Cape Town, South Africa, using convenience sampling. At these sites, the study was advertised via pamphlets, emails, or visual electronic representations on plasma screens. The study included a total of 93 participants, 40 with current SAD with childhood trauma (moderate/severe), 22 with current PTSD secondary childhood trauma (moderate/severe) (*further referred to as SAD and PTSD, respectively*), and 31 with no psychiatric disorder and no childhood trauma (i.e., healthy controls).

The mean age of participants (*N* = 93) was 34 years (*SD* = 11; range of 20–72 years). Most were *female* (55.9%, *n* = 52), *Caucasian* (58.1%, *n* = 54), 25.8% (*n* = 24) of mixed race [Colored], Black (12.9%, *n* = 12), Asian (2.2%, *n* = 2), and other (1.1%, *n* = 1). 52 were *single* (55.9%), married (23.7%, *n* = 22), living with a partner (11.8%, *n* = 11), divorced (6.5%, *n* = 6) or widowed (2.2%, *n* = 2). The majority were employed (63.4%, *n* = 59), had 12 or more years of education (83.8%, *n* = 78), and had an annual household income of more than R60 000 per annum ($6,000) (76.3%, *n* = 71).

Participants were assessed with the Social Anxiety Scale (LSAS), Clinician-Administered PTSD Scale (CAPS), Childhood Trauma Questionnaire—Short Form (CTQ-SF, *further referred to as the CTQ*), Connor-Davidson Resilience Scale (CD-RISC), and the Mini International Neuropsychiatric Interview (MINI) version 6.0 (Sheehan et al., 1998) to determine whether they met inclusion criteria for one of the three groups. These assessments were administered by a clinical psychologist. On the basis of this screening, participants were categorized into the following groups: PTSD, SAD, and controls. Both right and left handed participants were included and were carefully matched across groups and handedness was accounted for in all statistical analyses. Other exclusion criteria included the following Axis I disorders: all DSM-IV psychotic disorders, bipolar mood disorders, obsessive-compulsive disorder, eating disorders and current alcohol or substance abuse or addiction disorders. Other Axis one disorders were only included if in remission or if not established as the principal presenting complaint on the MINI. All neurological disorders, included head injuries with a loss of consciousness at any point in life, counted as exclusion from the study. Other criteria for exclusion included reported drug abuse/dependence or alcohol abuse/dependence within the past 6 months (Cooney et al., 2006). Subjects were excluded if they were currently taking psychotropic medication, besides an SSRI. Due to the fMRI scanning component of the main study, participants were excluded if they also had a cardiac pacemaker, metal prosthesis or pin(s), clips on blood vessels, inner ear prosthesis, an infusion pump, a metal intra-uterine contraceptive device or they were currently pregnant. All metal objects and jewelry were removed before any scanning. With regard to the PTSD group, only participants with a diagnosis of PTSD that was secondary to childhood trauma were included, hence participants with PTSD secondary to adult-onset trauma were excluded.

### Measures

#### Diagnostics

SAD, PTSD and other psychiatric disorders were assessed with the MINI (Sheehan et al., 1998), based DSM-IV-R criteria (American Psychiatric Association, 2000). The MINI has good inter-rater and test-retest reliability (Lecrubier et al., 1997). Participants with psychotic disorders, personality disorders, neurological disorders, drug abuse/dependence (prescription and/or other) and alcohol abuse/dependence were excluded. Controls had no psychiatric disorders as assessed with the MINI and no childhood trauma exposure (as determined by a cut-off score of below 40 on the CTQ). For the purpose of MRI investigation, participants currently on psychotropic medication (with the exception of an SSRI), with a cardiac pacemaker, metal prosthesis or pin(s), clips on blood vessels, inner ear prosthesis, an infusion pump, a metal intra-uterine contraceptive device or pregnancy were excluded.

#### Social anxiety disorder

The LSAS is clinician-administered and consists of 24 questions (Baker et al., 2002), each on a 4-point Likert Scale. The cut-off score for clinically significant social anxiety is 60 (Safren et al., 1999), and is also used to discriminate between generalized and specific SAD (Baker et al., 2002). Examples of items include: “telephoning in public,” “writing while being observed,” and “meeting strangers.” A psychometric evaluation of the LSAS has shown good test-retest reliabilities of the total score (*r* = 0.83, *p* < 0.01) and fear/anxiety and avoidance sub scores (*r* = 0.79, *p* < 0.01, and *r* = 0.83, *p* < 0.01, respectively) (Baker et al., 2002). Cronbach alphas for the LSAS total score for this sample was 0.96. For the subscales, Cronbach alphas were 0.94 for fear/anxiety and 0.93 for avoidance, respectively.

#### Posttraumatic stress disorder

PTSD diagnosis was assessed with the CAPS. The CAPS can be used to identify current or lifetime PTSD. Cross-cultural research shows that the CAPS has high reliability with a coefficient alpha of 0.92 and strong convergent validity with instruments measuring depression, anxiety and levels of psychosocial functioning (Charney and Keane, 2007). Events include, amongst others, “natural disasters,” “fire or explosion,” and physical assault.” Participants are asked to check the appropriate boxes for each event, namely “happened to me,” “witnessed it,” “learned about it,” “not sure,” or “doesn't apply.” Frequency and intensity scores can also be determined (Blake et al., 1990; Weathers et al., 2001).

#### Childhood trauma

The CTQ is a retrospective self-report questionnaire of childhood trauma consisting of 28 items (i.e., trauma before the age of 18) (Bernstein and Fink, 1998). This scale measures five types of childhood trauma, namely physical abuse and neglect, sexual abuse, and emotional abuse and neglect (Bernstein and Fink, 1998). No trauma was indicated if a participant had a score of less than 40. To make a clear distinction between traumatized and non-traumatized individuals, all participants with scores between 41 and 46 were excluded (Bernstein et al., 1997). A score above 46 was used to delineate moderate/severe trauma (Bernstein and Fink, 1998). The CTQ has been found to have an internal consistency of 0.91 (Cronbach alpha). Furthermore, it has demonstrated good reliability: test-retest reliabilities ranging from 0.79 to 0.86 and internal consistency reliabilities ranging from a median of 0.66 for the physical neglect subscale to a median of 0.92 for the sexual abuse subscale (Bernstein and Fink, 1998). Moreover, results from a sample of racially mixed individuals showed internal consistency reliability rates for the entire measure (0.91) and four of the subscales. Within the current sample, the internal reliability (Cronbach alpha) of the CTQ total scale was 0.85. Cronbach alphas for the CTQ subscales were as follows: emotional Abuse (0.87), physical abuse (0.84), sexual abuse (0.91), emotional neglect (0.90), and physical neglect (0.67). Items include “I didn't have enough to eat,” “people in my family said hurtful or insulting things to me,” “someone molested me,” and “I had to wear dirty clothes.”

#### Resilience

Resilience was measured with the CD-RISC (Connor and Davidson, 2003). The CD-RISC is a self-report questionnaire that consists of 25 items. The scale asks participants about how much they agree with each statement, or if the situation has not occurred recently, and how they think they felt during the last month. Participants rate themselves on a 5-point Likert scale (ranging from 0 = “not at all true,” to 4 = “true nearly all of the time”) according to how much they agree or disagree with each statement (e.g., I am able to adapt when changes occur). The CD-RISC measures five constructs of resilience: firstly, personal competence and high standards; secondly, a person's instincts, person's capability of enduring negative emotional experiences; thirdly, positive acceptance of change and relationships that are secure. The fourth construct refers to control and the last to spiritual influences that may contribute to resilience. Psychometric evaluation of the CD-RISC conducted on clinical and general population samples found the scale to have good reliability (Cronbach α = 0.89), validity, psychometric properties, good internal consistency and test-retest reliability (*r* = 0.87) (Connor and Davidson, 2003). Within the current sample, the internal reliability (Cronbach alpha) of the CD-RISC was 0.94. Examples of items include the following: “I am able to adapt when changes occur,” “I tend to bounce back after illness, injury, or other hardships,” and “I give my best effort, no matter what the outcome may be.” The total CD-RISC scale is made up out of 5 factors. The reliability of these subscales in this sample was good: Factor 1; *notion of personal competence, high standards, and tenacity* (*r* = 0.90), Factor 2; *trust in one's instincts, tolerance of negative affect, and strengthening effects of stress* (*r* = 0.82), Factor 3; *positive acceptance of change, and secure relationships* (*r* = 0.76), Factor 4; *control* (*r* = 0.84), Factor 5; *spiritual influences* (*r* = 0.64).

### Ethical considerations

The study received approval from Stellenbosch University's Health Research Ethics Committee (HREC, Ethics number: N09/09/226, latest approval: 04/05/2014). Written informed consent was obtained from all participants by a clinical psychologist prior to the initiation of study procedures and participants were reimbursed for travel costs. A clinical psychologist judged whether participants were able to understand the aims and risks of the study and be able to provide written informed consent.

### Data analyses

The resilience total (CD-RISC) scores and separate resilience factor scores were normally distributed. Analysis of variance (ANOVA), followed by *post-hoc* Bonferroni testing, was undertaken to assess differences in resilience scores among the groups (SAD, PTSD, healthy controls). The CTQ total and subscale scores were not normally distributed. Non-parametric correlational analysis (Spearman's tests) of CD-RISC and CTQ subscale scores was performed for (1) PTSD and SAD (*n* = 62) and PTSD, SAD and Control (*n* = 92) groups. All tests were two-tailed and the significance level for Spearman's test was set at *p* = 0.0083 (0.05/6). All statistical analyses were conducted using IBM SPSS Statistics for Windows, Version 22.0. IBM Corp. Released 2013. Armonk, NY: IBM Corp. For an overview of the demographics and clinical data see Table 1. For an overview of the group means and standard deviations of the questionnaires see Table 2. For an overview of the ANOVA and *post-hoc* results see Tables 3, 4. For an overview of the correlations see Tables 5, 6.

**Table 1:** Demographic and clinical data.

***N* = 93**		**SAD + (*n* = 40)**		**PTSD + (*n* = 22)**		**HC (*n* = 31)**	
		***M***	***sd***	***M***	***sd***	***M***	***sd***
Age (y)		36.6	12.6	35.5	11.5	30	7.4
Gender (Males %)		45		40.9		45.2	
Years of education (y)^a^		14	2.3	12.8	3.4	15.8	3
Ethnicity (%)	African	10		22.7		9.7	
	Mixed race	30		36.4		12.9	
	Caucasian	55		40.9		74.2	
	Asian	2.5				3.2	
	Other	2.5					

*Results of sample; SAD+, Social anxiety with CHT; PTSD+, Posttraumatic stress disorder secondary to CHT; HC, Healthy controls. Demographic characteristics are presented in the first few rows, with each column representing the specific study group*.

a *Years of education starting from primary school through to tertiary education*.

**Table 2:** Means and standard deviations of the demographic variables years of education and age and the total and sub scores of psychopathology scores for the total sample, separate groups and traumatized vs. non-traumatized individuals.

**Groups**	**Total**		**SAD + (*n* = 40)**		**PTSD + (*n* = 22)**		**HC (*n* = 31)**	
***N* = 93**	***M***	***sd***	***M***	***sd***	***M***	***sd***	***M***	***sd***
LSAS_total	55.12	33.6	77.8	19.4	63.8	32.9	19.6	13.3
LSAS_A	26.6	16.9	37.9	10.5	31.1	15.8	8.8	7.1
LSAS_FA	28.5	17.4	39.9	10.3	32.6	17.6	10.8	7.6
CTQ_TT	12.6	6.1	60.3	13.4	61.6	15.1	30.4	5.1
CTQ_EA	12.6	6.0	16.3	4.1	14.6	5.8	6.3	1.7
CTQ_PA	8.8	4.5	9.9	4.6	10.6	5.5	6.0	1.5
CTQ_SA	8.0	4.9	8.7	5.3	9.9	5.9	5.6	1.7
CTQ_EN	12.9	5.8	15.8	4.7	15.8	4.3	6.9	2.4
CTQ_PN	8.4	3.7	9.5	3.6	10.5	3.7	5.5	1.2
CD_RISC_TT	66.4	18.1	58.2	17.2	60.8	15.4	80.7	11.1

*Total, results of sample as a whole; SAD+, Social anxiety with CHT; PTSD+, Posttraumatic stress disorder secondary to CHT; HC, Healthy controls; LSAS-Total, Liebowitz Social Anxiety Scale; LSAS-A, Avoidance subscale total score of the Liebowitz Social Anxiety Scale; LSAS-F, Fear subscale total of the Liebowitz Social Anxiety Scale; CTQ-TT, Childhood Trauma Questionnaire total score; CTQ_EA-,- PA, -SA, -EN, -PN, Childhood Trauma Questionnaire total scores of the subscales. Emotional Abuse, Physical Abuse, Sexual Abuse, Emotional Neglect, and Physical Neglect respectively, CD_RISC_TT, Connor Davidson Resilience Scale total score*.

**Table 3:** Bonferroni *post-hoc* adjustment of ANOVA between groups (PTSD, SAD, Controls).

**Participant group comparisons**		**Significance**	**Mean difference**	**95% Confidence interval**	
**Group 1 vs**.	**Group 2**			**Lower bound**	**Upper bound**
Controls vs.	SAD	0.000^***^	22.460	13.69	31.23
	PTSD	0.000^***^	19.846	9.63	30.06
SAD	PTSD	1.000	2.614	−12.34	7.11

*** *p < 0.001*.

**Table 4:** Bonferroni *post-hoc* adjustment of ANOVA between groups (PTSD, SAD, Controls).

**Participant group comparisons**		**Significance**	**Mean difference**	**95% Confidence Interval**	
**Group 1 vs**.	**Group 2**			**Lower bound**	**Upper bound**
**CD-RISC FACTOR 1**					
Controls vs.	SAD	0.000^***^	6.75	3.28	10.22
	PTSD	0.001^***^	6.04	2.00	10.09
SAD	PTSD	1.000	0.71	−4.56	3.14
**CD-RISC FACTOR 2**					
Controls vs.	SAD	0.000^***^	5.23	2.43	8.03
	PTSD	0.004^**^	4.41	1.14	7.67
SAD	PTSD	1.000	0.82	−3.93	2.28
**CD-RISC FACTOR 3**					
Controls vs.	SAD	0.000^***^	5.04	3.24	6.83
	PTSD	0.000^***^	4.80	2.71	6.88
SAD	PTSD	1.000	0.24	−2.23	1.75
**CD-RISC FACTOR 4**					
Controls vs.	SAD	0.000^***^	3.98	2.40	31.23
	PTSD	0.000^***^	3.41	1.58	30.06
SAD	PTSD	1.000	0.57	−2.31	7.11
**CD-RISC FACTOR 5**					
Controls vs.	SAD	0.018^*^	1.47	0.19	2.75
	PTSD	0.161	1.19	−0.30	2.68
SAD	PTSD	1.000	0.28	−1.69	1.14

* p < 0.05;

** p < 0.01;

*** *p < 0.001*.

**Table 5:** Spearman's correlations between resilience (CD-RISC) and childhood trauma as measured by the CTQ in the childhood trauma exposed group (SAD & PTSD) (*n* = 62).

**Variable**	**Spearman's rho (r value) and *p*-values**	**Emotional abuse (CTQ)**	**Emotional neglect (CTQ)**	**Physical abuse (CTQ)**	**Physical neglect (CTQ)**	**Sexual abuse (CTQ)**	**CTQ-Total score**
Resilience (CD-RISC)	*r*-value	0.110	−0.137	0.036	0.264	0.185	0.149
	*p*-value	0.396	0.287	0.779	0.038	0.149	0.248

^*^p < 0.000.

**Table 6:** Spearman's correlations between resilience (CD-RISC) and childhood trauma as measured by the CTQ in the combined group (SAD, PTSD, Controls) (*N* = 92).

**Variable**	**Spearman's rho (*r*-value) and *p*-values**	**Emotional abuse (CTQ)**	**Emotional neglect (CTQ)**	**Physical abuse (CTQ)**	**Physical neglect (CTQ)**	**Sexual abuse (CTQ)**	**CTQ-Total score**
Resilience (CD-RISC)	*r*-value	−0.358	−0.482	−0.220	−0.166	−0.060	−0.371
	*p*-value	0.000^*^	0.000^*^	0.035	0.113	0.567	0.000^*^

* *p < 0.0083*.

## Results

### Total resilience score

A main effect was found for total scores in resilience and group [*F*_(2.90)_ = 21.46, *p* < 0.001, η_*p*_^2^ = 0.32]. *Post-hoc* analyses revealed that the HC and SAD (*mean difference* = 22.5, *std error* = 3.6, *p* = 0.0001), and the HC and PTSD groups (*mean difference* = 19.8, *std error* = 4.2, *p* = 0.0001) differed significantly. The HC group was more resilient than the patient groups. The SAD and PTSD groups did not differ from each other (see Table 3).

#### Notion of personal competence, high standards, and tenacity

A main effect was found on factor 1 scores of the CD-RISC [*F*_(2.90)_ = 12.45, *p* < 0.001, η_*p*_^2^ = 0.22]. *Post-hoc* analyses revealed that the HC and SAD (*mean difference* = 6.7, *std error* = 1.4, *p* < 0.0001), and the HC and PTSD groups (*mean difference* = 6.0, *std error* = 1.6, *p* < 0.0001) differed significantly. The HC group was more resilient than the patient groups. The SAD and PTSD groups did not differ from each other (see Table 4).

#### Trust in one's instincts, tolerance of negative affect, and strengthening effects of stress

A main effect was found on factor 2 scores of the CD-RISC [*F*_(2.90)_ = 11.15, *p* < 0.001, η_*p*_^2^ = 0.20]. *Post-hoc* analyses revealed that the HC and SAD (*mean difference* = 5.2, *std error* = 1.1, *p* < 0.0001), and the HC and PTSD groups (*mean difference* = 4.4, *std error* = 1.3, *p* = 0.004) differed significantly. The HC group was more resilient than the patient groups. The SAD and PTSD groups did not differ from each other (see Table 4).

#### Positive acceptance of change, and secure relationships

A main effect was found on factor 3 scores of the CD-RISC [*F*_(2.90)_ = 26.94, *p* < 0.001, η_*p*_^2^ = 0.37]. *Post-hoc* analyses revealed that the HC and SAD (*mean difference* = 5.0, *std error* = 0.7, *p* < 0.0001) and the HC and PTSD groups (*mean difference* = 4.7, *std error* = 0.8, *p* < 0.0001) differed significantly. The HC group was more resilient than the patient groups. The SAD and PTSD groups did not differ from each other (see Table 4).

#### Control

A main effect was found on factor 4 scores of the CD-RISC [*F*_(2.90_) = 20.63, *p* < 0.001, η_*p*_^2^ = 0.31]. *Post-hoc* analyses revealed that the HC and SAD (*mean difference* = 3.9, *std error* = 0.6, *p* < 0.0001), and the HC and PTSD groups (*mean difference* = 3.4, *std error* = 0.7, *p* < 0.0001) differed significantly. The HC group was more resilient than the patient groups. The SAD and PTSD groups did not differ from each other (see Table 4).

#### Spiritual influences

A main effect was found on factor 5 scores of the CD-RISC [*F*_(2.90)_ = 4.16, *p* = 0.02, η_*p*_^2^ = 0.08]. *Post-hoc* analyses revealed that only the HC and SAD (*mean difference* = 1.5, *std error* = 0.5, *p* = 0.02), differed significantly. The HC group was more resilient than the SAD group. The SAD and PTSD groups, and the HC and PTSD groups did not differ from each other (see Table 4).

### Resilience and childhood trauma

No significant correlation was found between total resilience scores and childhood trauma scores in the childhood trauma (SAD and PTSD) groups (see Table 5). However, in the combined dataset (SAD, PTSD, healthy controls), significant negative correlations were found between resilience scores and the following: emotional abuse (*p* < 0.0083; *r* = −0.358), emotional neglect (*p* < 0.0083; *r* = −0.482), and total childhood trauma scores (*p* < 0.0083; *r* = −0.371) (see Table 6). We corrected for multiple comparisons using Bonferroni by dividing the *p*-value that we obtained (0.05) by the number of tests (6) that we ran for a particular analysis set. The adjusted *p*-value was set at 0.0083.

## Discussion

The current study is novel in that it examines the association between childhood trauma and resilience in individuals with PTSD and SAD compared with controls without childhood trauma. Congruent with our primary hypothesis, we found significant differences between PTSD, SAD, and controls respectively. Controls were more resilient compared to both disorder groups. This was true for all resilience factors (higher personal competence, higher standards, and greater reliance on own personal instincts [CD-RISC factor 1]; endurance of negative emotional experiences [CD-RISC factor 2]; more likely to positively accept change and make use of secure relationships when faced with stressors [CD-RISC factor 3]; greater control [CD-RISC factor 4]; greater use of spiritual influences to cope in a crisis [CD-RISC factor 5]). Several retrospective studies indicate that early exposure to trauma is associated in adulthood with an increased risk of developing anxiety and stress-related disorders (Kendler et al., 1995; Agid et al., 2000; Heim and Nemeroff, 2001; Spila et al., 2008), including PTSD (Ballenger et al., 2004; Bandelow et al., 2004; Etkin and Wager, 2007; Yehuda et al., 2010) and social anxiety disorder (SAD), (Heim and Nemeroff, 2001; Fossion et al., 2014). In addition, multiple trauma exposures in early life have been linked to increased PTSD symptoms (Collin-Vézina et al., 2011). Simon et al. (2009) hypothesized that childhood trauma (as measured with the total CTQ score) may be associated with less resilience. Specifically, emotional neglect was associated with less resilience in adults with SAD (Simon et al., 2009).

Further contrary to our second hypothesis we found no significant correlation between resilience scores and childhood trauma in the SAD and PTSD groups. When we conducted correlation analyses between resilience scores and childhood trauma in the combined dataset (PTSD, SAD, and controls), several significant negative correlations were found. These correlations were between resilience and the following: emotional abuse, emotional neglect, and total childhood trauma score. Not all individuals who experience childhood adversities develop PTSD, SAD or other psychopathology as adults (Collishaw et al., 2007) and may possess resiliency characteristics that protect them from developing psychopathology. Several factors have been associated with resilience, namely an internal locus of control, a sense of meaning, a strong self-esteem, and good problem-solving skills (Rutter, 1985; Taylor et al., 2000; Masten et al., 2009). In this paper resilience refers to personal competence, high standards, and instincts; capability of enduring negative emotional experiences; positive acceptance of change and secure relationships; control and spiritual influences (Connor and Davidson, 2003). In addition, the experience of childhood trauma in some individuals may contribute to their being less affected by stressors in adulthood (Campbell-Sills et al., 2006). Indeed, childhood trauma may lead individuals to become more resilient, or may enhance “steeliness” to future stressors in adulthood, even if they develop later psychopathology (Rutter, 2007), suggesting that resilience is more than just the counterpart of psychopathology and adversity (Ong et al., 2006).

However, in the face of early life adversity, outcomes can differ significantly from one individual to another (Masten et al., 1999; Hovens et al., 2012). These differences have been shown to be affected by the duration and severity of adversity as well as the timing of positive interventions (Horn et al., 2016). In addition, developmental timing of adversity, especially in infancy and adolescence, can impact differentially on lifelong trajectories of health and wellbeing (Gee and Casey, 2015).

This study adds to our understanding of the role of childhood trauma and resilience in individuals with PTSD, SAD and controls. There has been little comparative investigation of resilience in adults with an anxiety disorder and early childhood trauma exposure, specifically in SAD and PTSD. It is plausible that childhood trauma may interact with the level of resilience in adults with PTSD and SAD. To our knowledge there are no studies that have assessed resilience in PTSD and SAD in the context of childhood trauma. This is important given that resilience, against a background of childhood trauma, may be a modifiable target for treatment recovery in patients with anxiety and stress-related disorders. Reliability coefficients of the CTQ and CD-RISC measurements indicate high internal consistency (Cronbach alphas ≥0.85). All CTQ subscales had high Cronbach alphas (≥0.84), with the exception of physical neglect for which internal consistency was questionable (= 0.67).

A few limitations of this study warrant mention. Firstly, the use of the CD-RISC may be seen as a limitation. The CD-RISC only measures certain aspects of resilience, yet resilience is a complex (multidimensional) construct (Connor and Davidson, 2003). Connor and Davidson (2003) have also pointed out the need for more comprehensive, acceptable and well-validated measures of resilience that are easy to administer. An exploration of the resilience literature points out that there is still much heterogeneity in outcome (Rutter, 2007). The ongoing debate surrounding conceptual issues of resilience such as distinctions between promotive and vulnerability factors, the lack of studies examining gene x environment interactions, and research focusing on resilience on outcome variables alone instead of underlying processes, should be taken into consideration in future research, and point to the limitations of the present study (Luthar et al., 2006). Furthermore, with regards to positive change or a “steeling effect” following adversity exposure, Brooks et al. (2016) reported that age at event, active coping, PTSD symptom severity and social support may predict a positive vs. a negative change in resilience. In our opinion a more comprehensive assessment of resilience, childhood trauma severity and multiplicity, and other environmental stressors may provide a better understanding of contributory factors that may facilitate or hinder adaptation in individuals with SAD or PTSD. Second, the results of non-parametric correlational analysis should be cautiously interpreted in view of the small sample. Third, the sample was not representative of all ethnic groups in South Africa. Future studies will need to include Black South African and mixed race participants. Lastly, we collected childhood trauma data retrospectively from participants and older participants may have had more difficulty remembering childhood experiences (Spila et al., 2008). That said, it has been suggested that individuals tend to under-report childhood adversities (Hardt and Rutter, 2004).

In conclusion, this study provides preliminary data on the assessment of resilience in SAD and PTSD in the context of childhood trauma. Detailed investigation of resilience in these disorders, in larger samples of patients from different ethnic groups, is needed. Patients who have PTSD and SAD with moderate/severe childhood trauma appear to be significantly less resilient than those with no disorder. Assessing and addressing resilience in these disorders, particularly when childhood trauma is present may facilitate long-term recovery and warrants further investigation.

## Author contributions

MM: Has made substantial contributions to the conception and design, acquisition of data, and analysis and interpretation of data. Has been involved in drafting the manuscript and revising it critically for important intellectual content. SY and DR: Has made substantial contributions to the conception and design, acquisition of data, and interpretation of data. Has been involved in revising the manuscript critically for important intellectual content. JH: Has made substantial contributions to the statistical analysis and interpretation of data. Has been involved in revising the manuscript critically for important intellectual content. SS: Has made substantial contributions to the conception and design, analysis and interpretation of data. Has been involved in drafting the manuscript and revising it critically for important intellectual content. Provided final approval of the version to be published. All authors have read and approved the final manuscript.

### Conflict of interest statement

The authors declare that the research was conducted in the absence of any commercial or financial relationships that could be construed as a potential conflict of interest.
